# Bmi1 regulates auditory hair cell survival by maintaining redox balance

**DOI:** 10.1038/cddis.2014.549

**Published:** 2015-01-22

**Authors:** Y Chen, L Li, W Ni, Y Zhang, S Sun, D Miao, R Chai, H Li

**Affiliations:** 1 Department of Otorhinolaryngology, Hearing Research Institute, Affiliated Eye and ENT Hospital of Fudan University, Shanghai 200031, China; 2 Central Laboratory, Affiliated Eye and ENT Hospital of Fudan University, Shanghai 200031, China; 3 Institutes of Biomedical Sciences, Fudan University, Shanghai 200032, China; 4 State Key Laboratory of Reproductive Medicine, Research Center for Bone and Stem Cells, Department of Human Anatomy, Nanjing Medical University, Nanjing 210096, China; 5 Co-innovation Center of Neuroregeneration, Key Laboratory for Developmental Genes and Human Disease, Ministry of Education, Institute of Life Sciences, Southeast University, Nanjing 210096, China; 6 State Key Laboratory of Medical Neurobiology, Fudan University, Shanghai, China

## Abstract

Reactive oxygen species (ROS) accumulation are involved in noise- and ototoxic drug-induced hair cell loss, which is the major cause of hearing loss. Bmi1 is a member of the Polycomb protein family and has been reported to regulate mitochondrial function and ROS level in thymocytes and neurons. In this study, we reported the expression of Bmi1 in mouse cochlea and investigated the role of Bmi1 in hair cell survival. Bmi1 expressed in hair cells and supporting cells in mouse cochlea. Bmi1^−/−^ mice displayed severe hearing loss and patched outer hair cell loss from postnatal day 22. Ototoxic drug-induced hair cells loss dramatically increased in Bmi1^−/−^ mice compared with that in wild-type controls both *in vivo* and *in vitro*, indicating Bmi1^−/−^ hair cells were significantly more sensitive to ototoxic drug-induced damage. Cleaved caspase-3 and TUNEL staining demonstrated that apoptosis was involved in the increased hair cell loss of Bmi1^−/−^ mice. Aminophenyl fluorescein and MitoSOX Red staining showed the level of free radicals and mitochondrial ROS increased in Bmi1^−/−^ hair cells due to the aggravated disequilibrium of antioxidant–prooxidant balance. Furthermore, the antioxidant *N*-acetylcysteine rescued Bmi1^−/−^ hair cells from neomycin injury both *in vitro* and *in vivo*, suggesting that ROS accumulation was mainly responsible for the increased aminoglycosides sensitivity in Bmi1^−/−^ hair cells. Our findings demonstrate that Bmi1 has an important role in hair cell survival by controlling redox balance and ROS level, thus suggesting that Bmi1 may work as a new therapeutic target for the prevention of hair cell death.

Hearing loss is one of the most common sensory disorders in humans. Hair cells in the inner ear have an essential role in converting mechanical sound movement to neural signals for hearing and balance. Previous studies have reported that several genes involved in the survival of hair cells, including Pou4f3 (Xiang *et al.*^1^), Barhl1 (Li *et al.*^2^), Gfi1 (Wallis *et al.*^3^), Rb1 (Sage *et al.*^4^), Tmprss3 (Fasquelle *et al.*^5^), Eya1 (Lozlowki *et al.*^6^) and AMPK.^7^ Deficiency of these genes in hair cells lead to the loss of hair cells via lack of neurotrophic factors,^8^ disorder of cell cycle^4^ and dysfunction of cellular energy production.^7^ Reactive oxygen species (ROS) have important roles in noise- and ototoxic drug-induced hair cell damage and hearing loss.^9^ ROS include less-reactive ROS and high ROS (hROS); high levels of both less-reactive ROS and hROS can oxidize cell constituents, including DNA, proteins and lipids and active multiple apoptosis pathways including mitogen-activated protein kinase, Fas-FasL, NF-*κ*B, DNA damage response (DDR) and p53 signaling pathways.^10, 11, 12, 13^ ROS can also directly attack mitochondrion and lead to the release of cytochrome *c*.^14, 15^ Mitochondria are not only cellular organelles required for aerobic respiration but also the main source of intracellular ROS, which are thought to be the cause of most oxidative damage. Because of the abundant number of mitochondria and high consumption of oxygen, auditory hair cells are exposed to high level of oxidative stress, especially when challenged by ototoxic drugs.^9, 16, 17, 18, 19^ The balance between prooxidant and antioxidant molecules is critical to determine the rate of oxidative damage accumulation. Administration of ROS-scavenging antioxidants,^16, 20, 21, 22, 23^ as well as inhibition of oxidase,^18^ can reduce the ROS production, thus attenuating the subsequent hair cell death in ototoxic drug-treated cochleae.

Bmi1 is a member of the Polycomb group (PcG) family. PcG proteins form large complexes, including PRC1 (Polycomb repressive complex 1) and PRC2 (Polycomb repressive complex 2), which silence target genes by modifying chromatin organization.^24, 25^ Bmi1 also can bind to the Runx1/CBF*β* transcription factor complex to silence target gene in a PRC2-independent manner.^26^ Although many researchers focus on the role of Bmi1 in self-renewal of embryonic, adult and cancer stem cell,^27, 28, 29, 30, 31, 32, 33, 34, 35, 36^ several evidences suggest that Bmi1 also regulate cell survival by controlling mitochondrial function and ROS level. Previous report showed that Bmi1-deficient thymocytes have impaired mitochondrial function, which lead to a marked increase of intracellular ROS levels and subsequent engagement of the DDR pathway.^13^ In Bmi1-deficient CD34(+) stem cells, the reduced ability of self-renewal is associated with enhanced apoptosis, which coincided with increased levels of intracellular ROS.^37^ Bmi1 controls memory CD4 T-cell survival through direct repression of *Noxa* gene in an Ink4a- and Arf-independent manner.^38^ Overexpression of Bmi1 *in vivo* protects human embryonic stem cells (HSCs) from ROS damage and extends the lifespan of HSCs,^39^ whereas Bmi1 transduction *in vitro* reduced irradiation-induced ROS levels by suppressing the oxidase genes, including lactoperoxidase (Lpo), and increased repair of DNA damage in human keratinocytes.^40^

Bmi1 also expressed in terminally differentiated cells, such as neurons,^41^ besides stem cells and rapidly dividing cells. It is reported that Bmi1 is required in neurons to suppress p53-induced apoptosis via regulating the antioxidant defensive response.^42^ High Bmi1 expression level in cortical neurons resulted in the suppression of ROS through activation of antioxidant genes and conferred robust protection against DNA-damage-induced cell death or mitochondrial poisoning.^41^

However, the expression of Bmi1 and its function in the inner ear have not been reported. In this study, we investigated Bmi1 expression in mouse cochlea and its role in hair cell survival. We found that Bmi1 is expressed in the hair cells and supporting cells, and can regulate the redox balance and ROS levels, thus having an important role in the survival and sensitivity to ototoxic drug of auditory hair cells in mice cochleae.

## Results

### Bmi1 expressed in auditory hair cells

**Figure 1:**
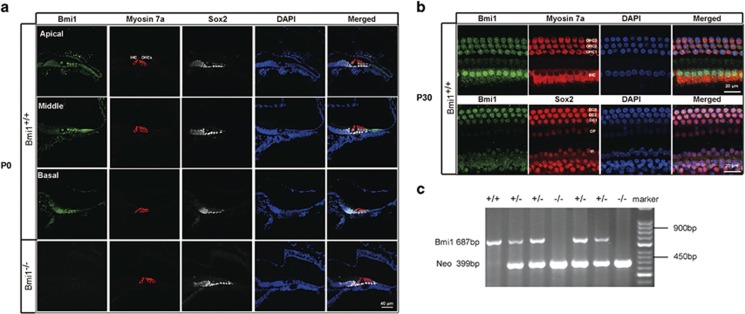
Bmi1 expressed in auditory hair cells and supporting cells. (**a**) Immunofluorescence staining showed Bmi1 expression in the apical, middle and basal turns in the Corti's organ of neonatal (P0) WT mice. Myosin 7a and Sox2 were used as hair cell and supporting cell markers, respectively. (**b**) Bmi1 expressed in the cochlear epithelium of P30 WT mice. (**c**) Typical PCR data of genotyping. Scale bars: 40 *μ*m (**a**); 20 *μ*m (**b**). OHC, outer hair cell; IHC, inner hair cell; DC, Deiters' cell; IP, inner pillar cell; OP, outer pillar cell

To investigate the Bmi1 expression in mouse cochlea, we used immunofluorescence staining with anti-Bmi1 antibody (Millipore, Consett, UK). Myosin 7a and sex-determining region Y)-box 2 (Sox2) were used as hair cell and supporting cell markers, respectively. Bmi1 expressed in both hair cells and supporting cells in the cochlea of neonatal and P30 wild-type (WT) mice (Figures 1a and b). Bmi1 also expressed in spiral ligament and spiral ganglion cells (data not shown).

### Bmi1^−/−^ mice showed severe hearing loss and auditory hair cell loss

**Figure 2:**
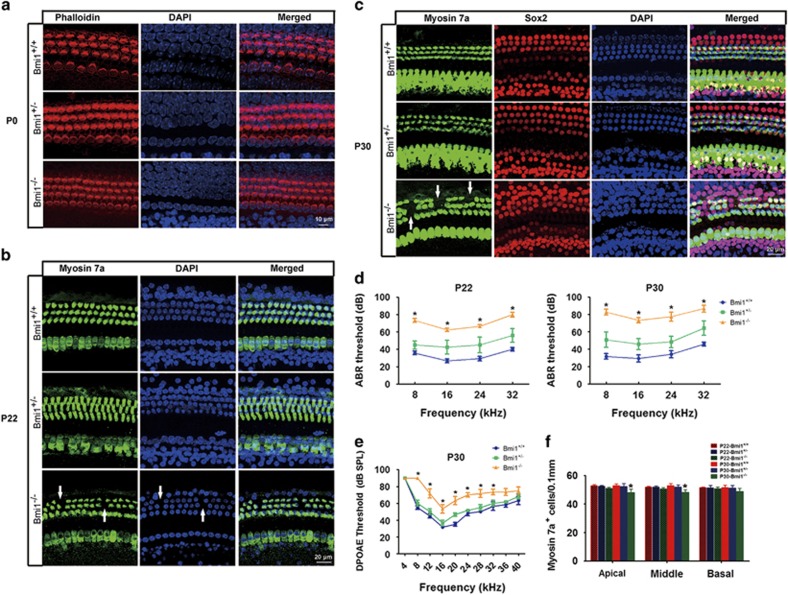
Bmi1^−/−^ mice showed severe hearing loss and patched auditory hair cell loss. (**a**) Phalloidin staining showed that the number and morphology of cochlear hair cells are similar among neonatal Bmi1^+/+^, Bmi1^+/−^ and Bmi1^−/−^ mice. (**b**) Myosin 7a staining showed dot loss of outer hair cells in P22 Bmi1^−/−^ mice. (**c**) Myosin 7a and Sox2 immunofluorescence staining showed patched loss of outer hair cells in P30 Bmi1^−/−^ mice. (**d**) ABR measurement revealed that hearing threshold at 8, 16, 24 and 32 KHz significantly increased in Bmi1^−/−^ mice compared with WT littermate at P22 and P30. (**e**) DPOAE measurement showed the function of outer hair cells was impaired in P30 Bmi1^−/−^ mice. (**f**) Statistical data of myosin 7a positive cells in the cochlea of P22 and P30 mice. Scale bars: 10 *μ*m (**a**); 20 *μ*m (**b** and **c**). *P<0.05 *versus* Bmi1^+/+^ group. *n*=6 for each group

Taking advantage of Bmi1 knockout (KO) (Bmi1^−/−^) mice, we investigated the role of Bmi1 in cochlear hair cells. Immunofluorescence and PCR results confirmed that Bmi1 did not express in the cochlea of Bmi1^−/−^ mice (Figures 1a and c). Auditory brain stem response (ABR) measurement revealed that hearing threshold at 8, 16, 24 and 32 KHz significantly increased in postnatal day 22 (P22) and postnatal day 30 (P30) Bmi1^−/−^ mice compared with WT littermate (Figure 2d) (*n*=6, *P*<0.05), indicating severe hearing loss in Bmi1^−/−^ mice. Distortion product otoacoustic emission (DPOAE) measurement showed that hearing threshold at 8, 12, 16, 20, 24, 28 and 32 KHz significantly increased in P30 Bmi1^−/−^ mice compared with WT littermate (Figure 2e) (*n*=6, *P*<0.05), suggesting that Bmi1 is important to hearing function. The hearing threshold of Bmi1^+/−^ mice showed no significant differences when compared with WT controls.

Morphological study showed there was no obvious change of cochlea structure at all three turns in the neonatal Bmi1^−/−^ mice. The number and morphology of hair cells in neonatal Bmi1^−/−^ mice is similar to that in WT littermate (Figure 2a), suggesting that Bmi1 does not affect the development of auditory hair cells. However, at P22, outer hair cell loss started to appear in the apical and middle turns of Bmi1^−/−^ mice (Figure 2b). More outer hair cell loss has been observed in all three turns at P30 (Figure 2c). Although supporting cells in Bmi1^−/−^ mice were also disorganized at P30, the number of supporting cells showed no significant difference among Bmi1^−/−^, Bmi1^+/−^ and WT mice (Figure 2c).

### Hair cells of Bmi1^−/−^ mice were more vulnerable to ototoxic drug-induced ototoxicity

**Figure 3:**
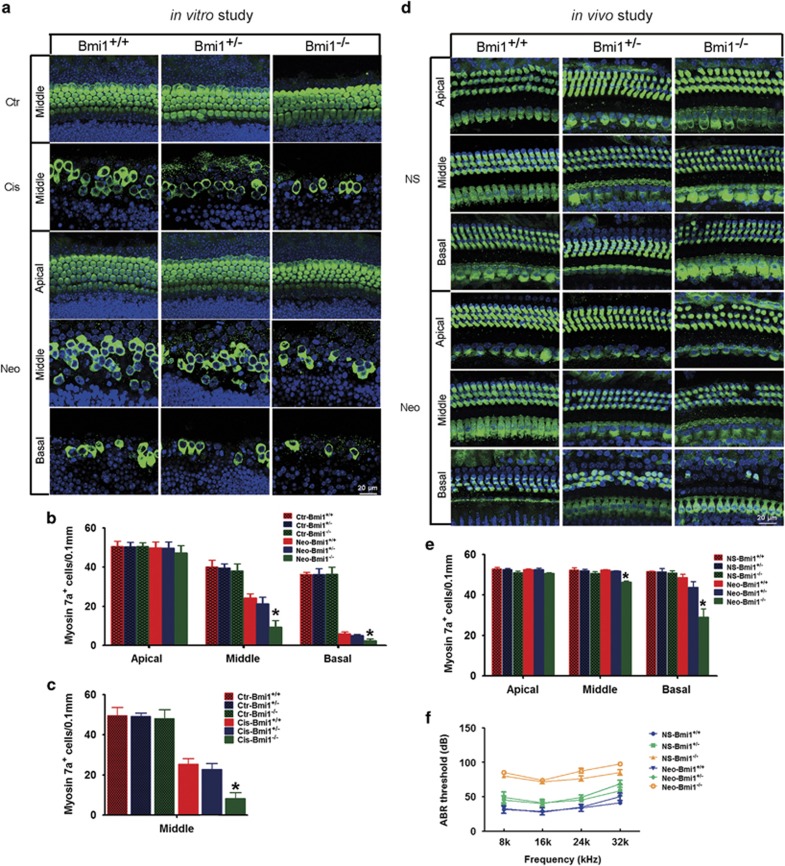
The sensitivity to ototoxic drugs increased in Bmi1^−/−^ auditory hair cells. (**a**) In the absence of damage, myosin 7a immunostaining showed that hair cells in cultured cochlear epithelium of newborn Bmi1^−/−^ mice were indistinguishable when compared with Bmi1^+/−^ and WT mice. After 10 *μ*M cisplatin or 0.25 mM neomycin treatment for 24 h, hair cell loss significantly increased in Bmi1^−/−^ cultured cochlear epithelium. Statistical data of myosin 7a-positive cells after neomycin (**b**) or cisplatin (**c**) treatment. (**d**) After treatment with neomycin (125 mg/kg/day for 5 days from P7), hair cell loss increased in Bmi1^−/−^ mice compared with that in Bmi1^+/−^ and Bmi1^+/+^ mice. (**e**) Statistical data showed that neomycin-induced cochlear hair cell loss significantly increased in Bmi1^−/−^ mice compared with control littermates. (**f**) Hearing threshold evaluated by ABR measurement of Bmi1^−/−^, Bmi1^+/−^ and Bmi1^+/+^ mice after neomycin treatment. Scale bars: 20 *μ*m. *P<0.05 *versus* Neo-Bmi1^+/+^ group in **b** and **e**, or Cis-Bmi1^+/+^ group in **c**. *n*=5 for each group

To investigate the role of Bmi1 in hair cell survival, first the cochlea of P0 Bmi1^−/−^, Bmi1^+/−^ and WT mice were cultured and treated with 0.25 mM neomycin for 24 h *in vitro*. In the absence of damage, hair cells in the cultured cochlea were indistinguishable between Bmi1^−/−^, Bmi1^+/−^ and WT mice (Figure 3a). After treatment with 0.25 mM neomycin for 24 h, hair cell loss in the apical, middle and basal turns of WT mice were 1.45±2.89%, 39.50±2.66% and 83.43±1.15%, respectively. Strikingly, in Bmi1^−/−^ mice, hair cell loss in the apical, middle and basal turns significantly increased to 6.73±3.64%, 73.51±4.49% and 93.39±1.16%, respectively (Figure 3a) (*n*=5, *P*<0.05). Again, hair cell loss in Bmi1^+/−^ mice showed no significant differences when compared with the WT controls. To further confirm this finding, we used another ototoxic drug, cisplatin, to investigate the injury sensitivity of Bmi1^−/−^ hair cells. Unlike neomycin, cisplatin injured both hair cells and supporting cells in cochlear epithelium. Hair cell loss induced by cisplatin has no significant difference among the apical, middle and basal turns. In WT mice, hair cell loss of middle turn was 49.09±2.88% after 10 *μ*M cisplatin treatment for 24 h. Hair cell loss significantly increased to 82.95±3.02% in Bmi1^−/−^ mice (Figure 3a) (*n*=5, *P*<0.05).Furthermore, the increased injury sensitivity to aminoglycosides of Bmi1^−/−^ hair cells was also confirmed by *in vivo* study. Neomycin (125 mg/kg/day) was administrated to the P7 Bmi1^−/−^, Bmi1^+/−^ and WT mice for 5 days. Ten days after neomycin injection, hair cell loss in the apical, middle and basal turns of WT mice were 0.44±0.32%, 0.25±0.34% and 5.69±1.67%, respectively, whereas in Bmi1^−/−^ mice, these percentages significantly increased to 0.72±0.48%, 11.05±0.66% and 43.09±4.04%, respectively (Figure 3b) (*n*=5, *P*<0.05). All these results suggested that endogenous expression of Bmi1 has an important role in the injury sensitivity to ototoxic drugs and the survival of cochlear hair cells.

### Apoptosis and DNA damage was involved in the increase of ototoxic drug-induced hair cell loss in Bmi1^−/−^ cochlea

**Figure 4:**
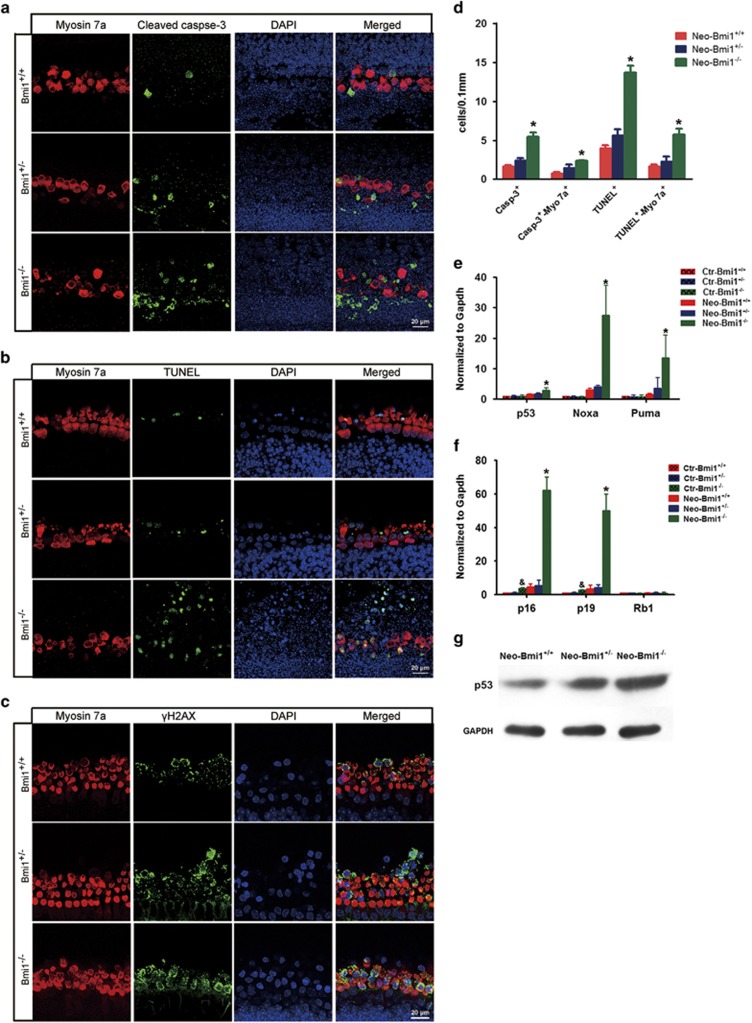
Apoptosis and DNA damage in hair cells was augmented in Bmi1^−/−^cochlear epithelium after neomycin insult. (**a**) Cleaved caspase-3 and myosin 7a double staining in Bmi1^−/−^, Bmi1^+/−^ and Bmi1^+/+^ cultured cochlear epithelium after neomycin treatment for 8 h. Middle turn. (**b**) TUNEL and myosin 7a double staining in Bmi1^−/−^, Bmi1^+/−^ and Bmi1^+/+^ cultured cochlear epithelium after neomycin treatment for 8 h. Middle turn. (**c**) *γ*H2AX and myosin 7a double staining in Bmi1^−/−^, Bmi1^+/−^ and Bmi1^+/+^ cultured cochlear epithelium after neomycin treatment for 8 h. Middle turn. (**d**) Statistical data revealed that the number of cleaved caspase-3^+^/myosin 7a^+^ and TUNEL^+^/myosin 7a^+^ cells significantly increased in Bmi1^−/−^ hair cells when compared with WT controls. (**e** and **f**) Real-time RT-PCR data showed the mRNA levels of p53, Noxa, Puma, p19, p16 and Rb1 after neomycin damage for 12 h. (**g**) Western blotting results showed the protein levels of p53 increased in Bmi1^−/−^ cochlear epithelium after neomycin damage for 12 h. Scale bars: 20 *μ*m. *P<0.05 *versus* Neo-Bmi1^+/+^ group. ^&^*P*<0.05 *versus* Ctr-Bmi1^+/+^ group. *n*=5 for each group

Previous studies revealed that apoptosis participate in the aminoglycoside-induced hair cell death.^43^ Cleaved caspase-3 and TUNEL could be used as markers of cell apoptosis. After neomycin treatment for 8 h, the numbers of caspase-3-positive and myosin7a/caspase-3 double-positive cells were significantly increased in Bmi1^−/−^ cochlea when compared with WT controls (Figure 4a). Increased apoptosis of Bmi1^−/−^ hair cells when treated with aminoglycosides was further confirmed by a similar increase in the number of TUNEL/myosin7a double-positive cells in Bmi1^−/−^ cochlear explants (Figure 4b).Quantitative RT-PCR and western blotting results showed that the expression of pro-apoptotic genes *p53* and p53 target genes, including *Noxa* and *Puma*, significantly increased in Bmi1^−/−^ hair cells (Figures 4e and g). Taken together, these data indicated that apoptosis involved in the increase of aminoglycosides induced hair cell loss in Bmi1^−/−^ mice.

*γ*H2AX (phosphorylated H2AX at the 139th serine residue), a sensitive indicator of DNA double-strand break, triggers Chk2 signaling pathway and p53 activation.^44^ After neomycin treatment, the level of *γ*H2AX significantly increased in Bmi1^−/−^ hair cells when compared with WT controls (Figure 4c), suggesting that DNA-damage-induced Chk2 signalling pathway might be involved in the increased sensitivity to aminoglycosides of Bmi1^−/−^ hair cells.

### Free radicals and mitochondrial ROS levels increased in Bmi1^−/−^ hair cells after neomycin insult

**Figure 5:**
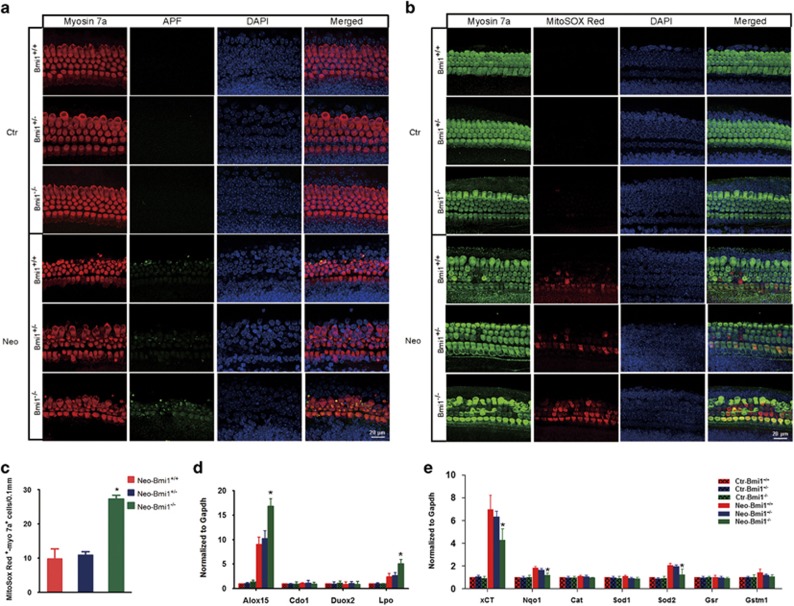
The level of ROS increased and the disequilibrium of antioxidant–prooxidant balance deteriorated in Bmi1^−/−^ hair cells after neomycin insult. (**a**) In the absence of damage, APF fluorescence in cochlear epithelium could not be detected. Two hours after 0.25 mM neomycin treatment, APF fluorescence is obviously stronger in hair cells of Bmi1^−/−^ mice compared with that in WT and Bmi1^+/−^ mice. Middle turn. (**b**) In the absence of damage, MitoSox Red fluorescence in cochlear epithelium could not be detected. Two hours after 0.25 mM neomycin treatment, MitoSox Red fluorescence is obviously stronger in hair cells of Bmi1^−/−^ mice, compared with that in WT and Bmi1^+/−^ mice. Middle turn. (**c**) Statistical data revealed that the number of MitoSox Red^+^/myosin 7a^+^ cells significantly increased in Bmi1^−/−^ hair cells when compared with WT controls. Quantitative data showed the expression level of antioxidant genes (**d**) and oxidases (**e**) at 2 h after neomycin treatment. Scale bars: 20 *μ*m. *P<0.05 *versus* Neo-Bmi1^+/+^ group. *n*=5 for each group

It has been reported that aminoglycosides induced accumulation of hROS is closely related to hair cell damage.^45^ Aminophenyl fluorescein (APF), which is a green probe, can selectively detect hROS, including free hydroxyl radical (•OH), peroxynitrite (ONOO−) and hypochlorite (−OCl). In the absence of damage, APF fluorescence in cochlear epithelium could not be detected in both Bmi1^−/−^ and WT mice (Figure 5a). Two hours after neomycin treatment, APF fluorescence could be detected in hair cells and the fluorescence intensity was significantly higher in Bmi1^−/−^ mice than that in WT control (Figure 5a), indicating that hROS level significantly increased in Bmi1^−/−^ mice. Auditory hair cells possess many mitochondria and consume large amount of oxygen, and thus are sensitive to oxidative stress. MitoSOX Red, a redox fluorophore detecting selectively mitochondrial superoxide,^13, 46, 47^ were used to evaluate mitochondrial ROS generation in hair cells. In the absence of damage, no MitoSOX Red fluorescence was detected in both Bmi1^−/−^ and WT epithelium (Figure 5b). Two hours after neomycin treatment, the fluorescence intensity of MitoSOX Red was significantly higher in Bmi1^−/−^ mice than that in WT control (Figure 5b), indicating that mitochondrial ROS level significantly increased in Bmi1^−/−^ mice. These results suggested that the increased levels of ROS might be the reason for the increased injury sensitivity to aminoglycosides of Bmi1^−/−^ hair cells.

### The disequilibrium of antioxidant–prooxidant balance deteriorated in Bmi1^−/−^ hair cells after neomycin injury

Cellular redox homeostasis depends on the antioxidant–prooxidant balance. The increment of ROS levels in Bmi1^−/−^ hair cells might be caused by the decreased antioxidant levels and/or the increased prooxidant levels. Previous studies have reported the decreased expression levels of antioxidant genes (*xCT*, *Nqo1*, *Sod1* and *Sod2*) in Bmi1-deficient neurons^42^ and the increased expression levels of oxidizing enzymes (Alox5, arachidonate 15-lipoxygenase (Alox15), Duox1, dual oxidase 2 (Duox2), cysteine dioxygenase 1 (Cdo1) and Lpo) in Bmi1-deficient thymocytes.^13^ To find out how Bmi1 deficiency leads to elevated ROS levels in Bmi1^−/−^ cochlea, quantitative RT-PCR was used to investigate the expression levels of several antioxidant (*xCT*, *Nqo1*, *Cat*, *Sod1*, *Sod2*, *Gsr* and *Gstm1*) and prooxidant genes (*Alox15*, *Lpo*, *Duox2* and *Cdo1*). In the absence of damage, the expression of both antioxidant and prooxidant genes showed no significant difference between Bmi1^−/−^ and WT neonatal mice (Figures 5d and e). Two hours after neomycin treatment, the expression levels of both antioxidant and prooxidant genes dramatically increased in the WT cochlea. However, the increment amplitude of prooxidant gene expression, including Alox15, Cdo1 and Lpo, increased significantly more in Bmi1^−/−^ cochlea than in WT control (Figure 5d), whereas the increment amplitude of antioxidant gene expression, including xCT (also known as Slc7a11, solute carrier family 7, member 11), Nqo1 (NAD(P)H dehydrogenase, quinone 1) and superoxide dismutase 2, mitochondrial (Sod2), increased significantly more in WT control than in Bmi1^−/−^ cochlea (Figure 5e). These data demonstrated that the disequilibrium of antioxidant–prooxidant balance was aggravated in Bmi1^−/−^ cochlear hair cells, which resulted in the much more severe ROS accumulation in Bmi1^−/−^ hair cells than in WT control.

### Antioxidant treatment rescued the hair cells in Bmi1^−/−^ cochlea

**Figure 6:**
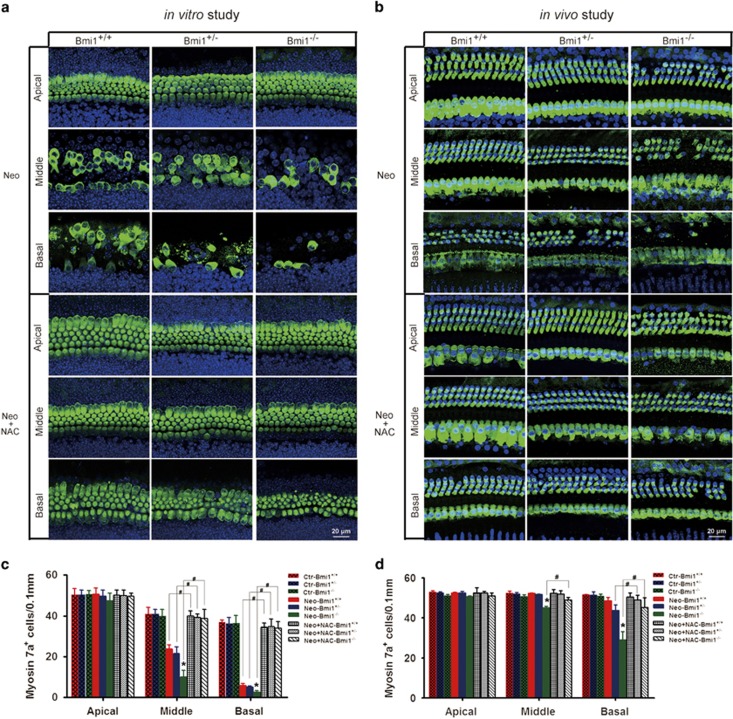
Antioxidant treatment rescued Bmi1^−/−^ hair cells. (**a**) *In vitro* study showed that NAC treatment rescued Bmi1^−/−^ hair cells from neomycin injury. (**b**) *In vivo* study showed that neomycin induced hair cells loss attenuated in Bmi1^−/−^ cochlea after neomycin treatment. (**c** and **d**) Statistical data of survival hair cells after neomycin and NAC treatment. Scale bars: 20 *μ*m. *P<0.05 *versus* Neo-Bmi1^+/+^ group; ^#^*P*<0.05. *n*=5 for each group

To further investigate whether the increment of ROS levels contribute to the increased injury sensitivity to aminoglycosides of Bmi1^−/−^ hair cells, the antioxidant *N*-acetylcysteine (NAC), which is a reduced glutathione (GSH) provider and a direct scavenger of reactive oxygen intermediates, was used to treat the neomycin-injured hair cells. After NAC (20 *μ*M) treatment, hair cell loss dramatically decreased in both Bmi1^−/−^ and WT control mice, and the number of survived hair cells has no significant difference between Bmi1^−/−^ and WT mice (Figures 6a and c). The rescue effect of antioxidant treatment was further confirmed by *in vivo* study (Figures 6b and d), demonstrating that ROS accumulation was the major cause of the high injury sensitivity of Bmi1^−/−^ auditory hair cells to aminoglycosides.

## Discussion

The Polycomb gene *Bmi1* is extensively studied in stem cells and rapid dividing cells, such as cancer cells and lymphocytes. Although the role of Bmi1 in stem cell renewal has been largely understood, several evidences suggest that Bmi1 also regulates cell survival by controlling mitochondrial function and ROS level. However, the expression and function of Bmi1 in the cochlea remains unclear. In this study, we reported the expression of Bmi1 and demonstrated that Bmi1 regulates the survival of hair cells by controlling the accumulation of ROS in postnatal mice cochlea. We reported the severe hearing loss (Figure 2), the daily-increasing hair cell loss from P22 (Figure 2) and the increased susceptibility of hair cells to ototoxic drugs (Figure 3) in Bmi1 KO mice, which indicated that Bmi1 has an important role in regulating the survival of auditory hair cells. After neomycin treatment, we found loss of Bmi1 led to the significant increased accumulation of free radicals and mitochondrial ROS (Figure 5), which induced DNA damage and apoptosis of hair cell (Figure 4); and antioxidant treatment rescued this hair cell loss in Bmi1 KO cochlea (Figure 6).

The plenty of mitochondria and high oxygen consumption in mouse auditory hair cells make them sensitive to oxidative stress especially challenged by external stimulation such as noise and ototoxic drugs.^9^ It has been reported that Bmi1-deficient thymocytes^13^ and neurons^42^ have an increased level of intracellular ROS due to deregulated expression of genes related to mitochondrial function and ROS generation. In our study, we reported severe ototoxic drug-induced hair cell damage (Figure 3) accompanied with higher levels of free radicals and mitochondrial ROS accumulation (Figure 5) in Bmi1^−/−^ hair cells after neomycin injury, which suggested ROS involved in the increased injury sensitivity to aminoglycosides in Bmi1^−/−^ cochlea. After antioxidant NAC treatment, the number of survived hair cells in Bmi1^−/−^ mice had no significant difference compared with WT mice (Figure 6). This finding demonstrated that the high ROS level was the major cause of the high injury sensitivity to ototoxic drugs in Bmi1^−/−^ cochlea, and indicated that Bmi1 regulates the hair cell survival by controlling the levels of ROS.

Balance between prooxidant and antioxidant molecules is critical to determine the rate of oxidative damage accumulation. When external stimulation brings out accumulation of ROS, the expression of antioxidant response genes also increased. In this study, we first reported the injury-induced dramatic increase of oxidizing enzymes (Alox15 and Lpo) and antioxidant genes (*xCT*, *Nqo1* and *Sod2*) in mouse cochlea (Figure 5). Previous studies have reported the increased expression of oxidizing enzymes in Bmi1^−/−^ thymocytes^13^ and decreased expression of antioxidant enzymes in Bmi1^−/−^ neurons.^42^ In Bmi1^−/−^ cochlea, the increment amplitude of oxidizing enzymes increased, while the increment amplitude of antioxidant genes decreased compared with WT control after neomycin injury (Figure 6), indicating that aggravated disequilibrium of antioxidant–prooxidant balance is responsible to the increased accumulation of ROS. These results suggested that Bmi1 controls the antioxidant–prooxidant balance to maintain redox homeostasis in mouse auditory hair cells.

Several pathways are involved in Bmi1 deficiency-induced cell death, including the following: (1) Bmi1 deficiency leads to mitochondrial dysfunction resulting in increased levels of intracellular ROS and subsequent engagement of the DDR pathway in which Chk2 and p53 were activated;^13^ (2) Bmi1 deficiency directly leads to p53-mediated repression of antioxidant genes, resulting in increased ROS;^41^ (3) Bmi1 deficiency deregulates the expression of p19, resulting in phosphorylation of p53, which triggers p53-dependent apoptosis;^28^ (4) Bmi1 deficiency also can directly deregulate the expression of Noxa (also known as Pmaip1, phorbol-12-myristate-13-acetate-induced protein 1) independent of p19 and p53 (Yamashita *et al.*^38^). In the absence of neomycin injury, the expression of p16 and p19 had already significantly increased in cultured neonatal Bmi1^−/−^ cochlea, although there was no significant hair cell loss in neonatal Bmi1^−/−^ cochlea (Figure 4). After neomycin treatment, significantly more hair cell loss was observed in Bmi1^−/−^ cochlea when compared with WT control (Figure 3), and this was accompanied by significantly higher levels of free radicals and mitochondrial ROS (Figure 5), more DNA damage-induced *γ*H2AX foci (Figure 4), as well as higher expression levels of p16, p19, p53 and p53 target genes (*noxa* and *puma*) in Bmi1^−/−^ cochlea than in WT control (Figure 4). ROS can oxidize cell constituents, such as DNA, lead to DNA damage and activate multiple apoptosis pathways including p53-dependent apoptosis.^48, 49, 50^ *γ*H2AX triggers Chk2 signaling pathway and p53 activation. In cooperation with *γ*H2AX, p53 repairs DNA damage or induces cell death.^51^ In our study, we found that the peak of free radicals and mitochondrial ROS level was 2 h after neomycin treatment, whereas the peak of the expression of p53, noxa and puma were 12 h after neomycin treatment; thus, we speculated that Bmi1 deficiency led to the disequilibrium of antioxidant–prooxidant balance, resulting in increased accumulation of ROS, which then triggered DNA damage and multiple apoptotic pathways including p53-dependent apoptosis.

NAC, an essential precursor to many endogenous antioxidants involved in the decomposition of peroxides, attenuates oxidative stress by replenishing intracellular GSH stores.^52^ In clinical trials, NAC improves lung function in patients with chronic obstructive pulmonary disease, highlighting the potential benefit of ROS-directed therapy.^53^ In the current study, both *in vivo* and *in vitro* results confirmed that NAC treatment ameliorated neomycin-induced hair cell damage, suggesting that antioxidant treatment might be an effective therapy for hearing loss induced by ototoxic drugs. Furthermore, we demonstrated that the increased sensitivity to ototoxic drugs caused by Bmi-1 deficiency was largely rescued by antioxidant treatment, indicating that Bmi1 regulated hair cell survival and sensitivity to ototoxic drugs by maintaining redox balance.

In summary, we first reported the expression pattern of Bmi1 in mouse cochlea and then demonstrated that Bmi1 has an important role in the hair cell survival and regulates the sensitivity of hair cells to ototoxic drug via controlling redox balance. Lastly, we approved that ROS accumulation was the main reason for increased aminoglycoside sensitivity in Bmi1^−/−^ hair cells, both *in vitro* and *in vivo*, via the antioxidant NAC rescue experiments. Our findings may provide new therapeutic targets for prevention of the hair cell death.

## Materials and Methods

### Mice and genotyping

Bmi1^+/−^mice (129Ola/FVB/N hybrid background) that were backcrossed 10–12 times onto a C57BL/6J background were mated to generate Bmi1^−/−^, Bmi1^+/−^ and WT mice (littermates) genotyped by PCR, as described previously.^27^ This study was carried out in strict accordance with the ‘Guiding Directive for Humane treatment of Laboratory Animals' issued by the Chinese National Ministry of Science and Technology on September, 2006. All experiments were approved by the Shanghai Medical Experimental Animal Administrative Committee (Permit Number: 2009-0082). All efforts were made to minimize suffering and reduce the number of animals used.

### Experimental protocol (*in vivo* studies)

To explore the effect of Bmi1 deficiency on injury sensitivity to ototoxic drug in cochlear hair cells, neomycin was injected subcutaneously once daily for 5 days from postnatal day 7 (P7) at a final dose of 125 mg/kg/day. After exposure to neomycin, the hearing threshold was evaluated by ABR measurement at P22. For NAC rescue experiment, NAC (200 mg/kg/day, i.p.) were injected for 7 days at the beginning of neomycin insult. Next, mice of either sex were killed and cochleae were fixed in 4% paraformaldehyde (PFA). After decalcification, cochlear epithelium was prepared for morphological analysis.

### ABR and DPOAE measurement

ABR analysis was performed in anesthetized P22 and P30 mice, to measure the hearing threshold, as described previously.^54^ The hearing threshold of P22 and P30 mice was assessed at four frequencies (8, 16, 24 and 32 kHz) in a TDT system 3 (Tucker-Davies Technologies, Gainesville, FL, USA). DPOAE signal was measured with a TDT-RZ6 system (Tucker-Davis Technologies). The stimuli for eliciting DPOAEs were two sine wave tones of differing frequencies (F2=1.2F1) of 1 s duration with F2 ranging from 4 to 40 kHz. The two tones were presented at identical intensities, which ranged from 20–90 dB SPL in 10 dB increments. The acoustic signal picked up by the microphone in the earbar was digitized at 200 kHz and the magnitude of the 2F1−F2 distortion product determined by FFT. The surrounding noise floor was also calculated by averaging 20 adjacent frequency bins around the distortion product frequency. DPOAE thresholds were calculated offline by interpolating the data and identifying when the signal was greater than −5 dB SPL and greater than two S.D. above the noise floor. If no DPOAE response was detected even at our equipment limits of 90 dB SPL, we arbitrarily defined the threshold to be 90 dB.

### Experimental protocol (*in vitro* studies)

Cochlear sensory epithelium was dissected from P0 mice and cultured as previously reported.^55^ Neomycin (0.25 mM, Sigma-Aldrich, St. Louis, MO, USA) or cisplatin (10 *μ*M, Sigma-Aldrich) was added for 24 h to kill hair cells. After neomycin or cisplatin was removed, the tissues were cultured in serum-free medium for additional 48 h. For NAC rescue experiment, NAC (20 mM) were added in medium for 48 h at the beginning of neomycin insult.

### Immunofluorescence, phalloidin, TUNEL and APF staining

Mouse monoclonal anti-Bmi1 (Millipore, 1 : 500), rabbit polyclonal anti-cleaved caspase-3 (s9661, Cell Signaling Technology Inc, Danvers, MA, USA), polyclonal anti-myosin VIIA (Myo7a, 1 : 1000) (Proteus Biosciences, Ramona, CA, USA), polyclonal anti-*γ*H2AX (Abcam, Cambridge, UK; 1 : 200), goat polyclonal anti-SR*γ* (sex-determining region *γ*)-box 2 (Sox2) (sc-17320, Santa Cruz Biotechnology, Dallas, TX, USA) and phalloidin–tetramethylrhodamine (TRITC) (Sigma-Aldrich, 1 : 1000) were used. Briefly, nonspecific binding sites were blocked for 1 h in 0.3% Triton X-100 and 10% (v/v) heat-inactivated normal serum in PBS (PBT1). Tissues were then incubated overnight at 4 °C in PBT1 with primary antibodies. After unbound antibodies were removed, tissues were incubated with the corresponding secondary antibodies conjugated with TRITC, fluorescein isothiocyanate or Cy5 (Jackson ImmunoResearch, West Grove, PA, USA). Counterstaining with DAPI (Sigma-Aldrich) allowed visualization of the cell nuclei. Specimens were examined by confocal fluorescence microscopy (Leica SP5, Heidelberg, Germany). Negative control experiments were performed as above by omitting the primary antibodies.

TUNEL Kit (Roche, Indianapolis, IN, USA) was used to detect apoptotic cells, according to the instructions of the manufacturer. APF staining was performed to detect hROS as previous reported.^45^ After being exposed in 0.25 mM neomycin, the explants were rinsed with HBSS and stained with APF (12.5 *μ*M, Cell Technology Inc., Beverly, MA, USA) for 30 min at 37 °C in humidified air with 5% CO_2_. APF is minimally fluorescent; however, when reacted with hROS (•OH, ONOO− and −OCl), they are converted to fluorescein and exhibit strong, dose-dependent fluorescence. MitoSOX Red (Invitrogen, Carlsbad, CA, USA) was used to detect mitochondrial ROS. After being exposed in 0.25 mM neomycin, the explants were washed with PBS and stained with MitoSOX Red probe (Invitrogen, 5 *μ*M), incubated for 10 min. After staining, the cells were fixed in PFA.

### Real-time PCR

Two micrograms of total RNA were used for reverse transcription with Superscript III reverse transcriptase (Invitrogen). Real-time PCR was performed on ABI 7500 real-time PCR system (Applied Biosystems, Foster City, CA, USA) using GoTaq qPCR Master Mix (Promega, Madison, WI, USA). All primers were designed to flank individual exons and tested by PCR. The optimized conditions were held constant for each sample to assure valid comparison of the results. Primers sets used were as follows: GAPDH(F) 5′-TGCGACTTCAACAGCAACTC-3′ (R) 5′-CTTGCTCAGTGTCCTTGCTG-3′, p16(F) 5′-CAACGCCCCGAACTCTTTC-3′ (R) 5′-ATCTGCACCGTAGTTGAGCA-3′, p19(F) 5′-TGTTGTTGAGGCTAGAGAGGA-3′ (R) 5′-CGAATCTGCACCGTAGTTGA-3′, p53(F) 5′-CATCCTCCTCCTTCCCAGC-3′ (R) 5′-CAGTGAGGTGATGGCAGGAT-3′, Rb1(F) 5′-AAGTTCTCACCTCCTGCACT-3′ (R) 5′-CTCTTCTGGGTGTTCGAGGT-3′, Noxa(Pmaip1)(F) 5′-AGTCGCAAAAGAGCAGGATG-3′ (R) 5′-AAGCTTTGGAAGAACTCAGGT-3′, Puma(also known as Bbc3, BCL2-binding component 3)(F) 5′-GTGTGGAGGAGGAGGAGTG-3′ (R) 5′-CTGGGTAAGGGGAGGAGTC-3′, superoxide dismutase 1, soluble (Sod1) (F) 5′-GGGTTCCACGTCCATCAGTA-3′ (R) 5′-GGTCTCCAACATGCCTCTCT-3′, Sod2(F) 5′-TGTTACAACTCAGGTCGCTCT-3′ (R) 5′-CTCCCACAGACACGGCTG-3′, xCT(F) 5′-TGGAGGTCTTTGGTCCTTTG-3′ (R) 5′-CCAGGATGTAGCGTCCAAAT-3′, catalase(F) 5′-AGCGGATTCCTGAGAGAGTG-3′ (R) 5′-GACTGTGGAGAATCGAACGG-3′, glutathione *S*-transferase, mu 1 (Gstm1) (F) 5′-TCCTGCCCACGTTTCTCTAG-3′ (R) 5′-AGTCTGTGTATTCCAGGAGCA-3′, Nqo1(F) 5′-ACTTCAACCCCATCATTTCCAG-3′ (R) 5′-TATCACCAGGTCTGCAGCTT-3′, glutathione reductase (Gsr) (F) 5′-TATGTGAGCCGCCTGAACA-3′ (R) 5′-GTGGCAATCAGGATGTGTGG-3′, Alox15(F) 5′-GACTTGGCTGAGCGAGGACT-3′ (R) 5′-CTTGACACCAGCTCTGCA-3′, Duox2(F) 5′-CCATCCTCAAAGACCTGGTCTTCA-3′ (R) 5′-CTCAGCCAGCTGAGTAATGTAGATGT-3′, Lpo(F) 5′-CTGGACCAGAAGAGATCCATG-3′ (R) 5′-TCACCAGGTGGGAACATGATGG-3′, Cdo1(F) 5′-GTGGATCAAGGAAATGGGA-3′ (R) 5′-CTTGATCATCTCGTTGGA-3′.

### Western blotting

Total protein was isolated using the AllPrep DNA/RNA/Protein Mini Kit (Qiagen, Valencia, CA, USA). Proteins were separated by 12% SDS-PAGE. After electrophoresis, the proteins were transferred to PVDF membranes (Millipore). The membranes were blocked with 5% non-fat dried milk in TBST for 1 h at room temperature. The primary antibodies, including anti-p53 antibody (Abcam, dilution 1: 1000) and anti-GAPDH antibody (Cell Signaling Technology Inc., dilution 1 : 1000), were added into the blocking buffer overnight at 4 °C. After rinsing three times (10 min each) with TBST, the membranes were incubated with HRP-conjugated secondary antibody at a concentration of 1 : 5000 (Supersignal West, Pierce, Rockford, IL, USA) for 1 h at room temperature. The immunoreactive bands were visualized using an ECL kit (Pierce).

### Cell counting and statistical analysis

To quantify the immunostaining positive cells, the entire cochlea was divided into nine segments of equal length from the apex to the base. Data are presented as the mean±S.D. ANOVA and Bonferroni's multiple comparison test were used to analyze the differences, and differences between groups were considered significant when *P*<0.05.

## Abbreviations

ROS: reactive oxygen species
APF: aminophenyl fluorescein
NAC: *N*-acetylcysteine
DDR: DNA damage response
PRC: Polycomb repressive complex
ABR: auditory brain stem response
DPOAE: distortion product otoacoustic emission
Alox15: arachidonate 15-lipoxygenase
Cdo1: cysteine dioxygenase 1
Lpo: lactoperoxidase
Duox2: dual oxidase 2
xCT: also known as Slc7a11, solute carrier family 7, member 11
Nqo1: NAD(P)H dehydrogenase, quinone 1
Sod1: superoxide dismutase 1, soluble
Sod2: superoxide dismutase 2, mitochondrial
Gsr: glutathione reductase
Gstm1: glutathione *S*-transferase, mu 1
Noxa: also known as Pmaip1, phorbol-12-myristate-13-acetate-induced protein 1
Puma: also known as Bbc3, BCL2-binding component 3
